# Pyrocurzerenone suppresses human oral cancer cell metastasis by inhibiting the expression of ERK1/2 and cathepsin S proteins

**DOI:** 10.1111/jcmm.70015

**Published:** 2024-08-19

**Authors:** Ming‐Ju Hsieh, Chia‐Chieh Lin, Hsin‐Yu Ho, Yi‐Ching Chuang, Yu‐Sheng Lo, Mu‐Kuan Chen

**Affiliations:** ^1^ Oral Cancer Research Center Changhua Christian Hospital Changhua Taiwan; ^2^ Graduate Institute of Clinical Medicine, College of Medicine National Chung Hsing University Taichung Taiwan; ^3^ Doctoral Program in Tissue Engineering and Regenerative Medicine, College of Medicine National Chung Hsing University Taichung Taiwan; ^4^ Graduate Institute of Biomedical Sciences China Medical University Taichung Taiwan; ^5^ Department of Otorhinolaryngology, Head and Neck Surgery Changhua Christian Hospital Changhua Taiwan

**Keywords:** cathepsin S, ERK, invasion, migration, oral, Pyrocurzerenone

## Abstract

Pyrocurzerenone is a natural compound found in *Curcuma zedoaria* and *Chloranthus serratus*. However, the anticancer effect of pyrocurzerenone in oral cancer remains unclear. Using the MTT assay, wound healing assay, transwell assay and western blot analysis, we investigated the impact of pyrocurzerenone on antimetastatic activity, as well as the critical signalling pathways that underlie the processes of oral cancer cell lines SCC‐9, SCC‐1 and SAS in this work. Our findings suggested that pyrocurzerenone inhibits cell migration and invasion ability in oral cancer cell lines. Furthermore, phosphorylation of ERK1/2 had significant inhibitory effects in SCC‐9 and SCC‐1 cell lines. Combining ERK1/2 inhibitors with pyrocurzerenone decreased the migration and invasion activity of SCC‐9 and SCC‐1 cell lines. We also found that the expressed level of cathepsin S decreased under pyrocurzerenone treatment. This study showed that pyrocurzerenone reduced ERK1/2 expression of the proteins and cathepsin S, suggesting that it could be a valuable treatment to inhibit human oral cancer cell metastasis.

## 1 INTRODUCTION

In Taiwan, oral cancer ranks sixth in terms of cancer‐related morbidity and death and second among those between the ages of 45 and 54 in 2020.^1^, ^2^, ^3^ Chewing betel nuts, smoking and drinking alcohol are some of the significant variables that lead to the development of oral cancer.^4^, ^5^ The 5‐year survival rate for oral malignancies in Taiwan is only approximately 53.9%, even though the disease can be treated with surgical resection, chemotherapy, radiation therapy or a combination of two or three of these techniques.^6^, ^7^

In human cancer cells, cathepsin S has been shown to participate in several processes, including angiogenesis, invasion and apoptosis.^8^, ^9^ Tsai et al. discovered that cathepsin S inhibitors may be beneficial in preventing or delaying cancer metastasis.^10^ The ERK pathway and cathepsin S are both involved in oral cancer cells. Previous studies have suggested that sulforaphane reversed oral cancer cell motility by inhibiting ERK1/2 pathway.^11^, ^12^

Pyrocurzerenone is an organic compound from the class of sesquiterpenoids, extracted from Chloranthus sertusra's herbs. However, the effect of pyrocurzerenone and its mechanism of action on oral cancer cells are still unknown. In this study, we examined pyrocurzerenone's antimetastatic effects of pyrocurzerenone on oral cancer cells. Our results suggested that pyrocurzerenone decreased oral cancer cell migration and invasion ability by inhibiting ERK1/2 activation and cathepsin S expression.

## 2 MATERIALS AND METHODS

### 2.1 Cell culture and reagents

**FIGURE 1:**
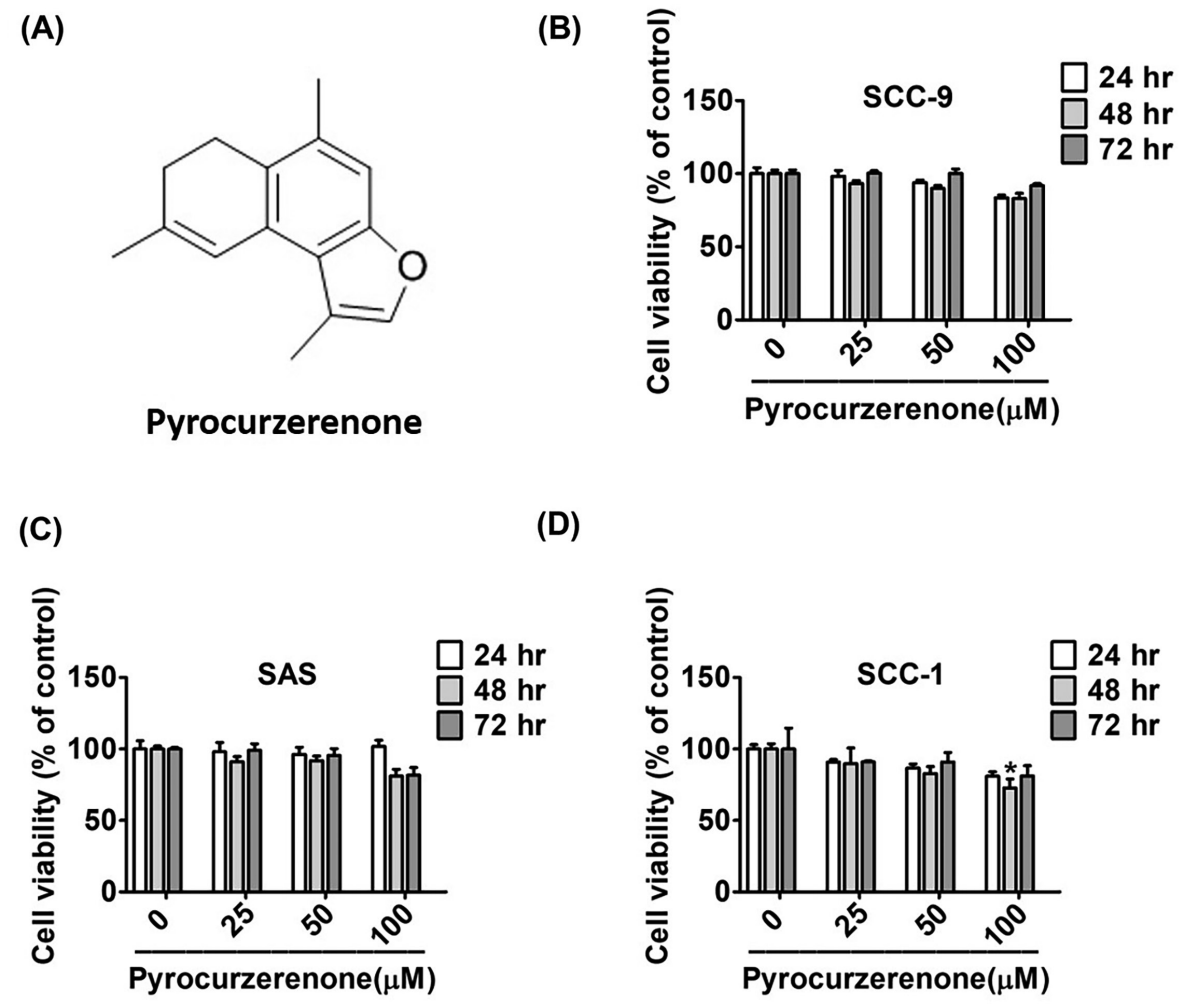
Non‐cytotoxicity of pyrocurzerenone at a dose less than 100 μM in oral cancer cell lines. (A) The chemical structure of pyrocurzerenone. (B–D) Cells were treated with pyrocurzerenone at different doses (0, 25, 50 and 100 μM) for 24 h. Cell viability was measured using the MTT assay. There was no difference between the vehicle group and the treatment group.

The oral cancer cell lines SCC‐9, SAS and SCC‐1 were purchased from the Japanese collection of the Research Bioresource Cell Bank (Shinjuku, Japan). All cell lines were grown in Dulbecco's modified eagle medium (DEME; Life Technologies, Grand Island, NY) supplemented with Ham's F12 nutritional mix (Life Technologies, Grand Island, NY), 10% fetal bovine serum (FBS), 1.2 g/L sodium bicarbonate, 15 mM HEPES and 1% penicillin/streptomycin. SCC‐9 cells were also treated with hydrocortisone (400 ng/mL) and 1% non‐essential amino acids (NEAA). All cells were kept at 37°C in a humidified atmosphere of 5% CO_2_. Pyrocurzerenone (≥98% purity; Figure 1A) was purchased from ChemFaces (Wuhan, Hubei, PRC). The powder of pyrocurzerenone was dissolved in dimethylsulfoxide (DMSO) at a concentration of 100 mM as a stock reagent. The total DMSO in the present experiments is less than 0.2%.

### 2.2 The cell viability assay

3‐ (4, 5‐dimethylthiazol‐2‐yl)‐2, 5‐diphenyltetrazolium bromide (MTT; MilliporeSigma) was used to detect cell viability.^13^ Briefly, after seeding 1 × 10^4^ cells/well of oral cancer cell lines in a 96‐well plate, different doses of pyrocurzerenone (25, 50 and 100 μM) were treated for 24 h. 0.1% DMSO was treated as a vehicle group (0 μM). Subsequently, the supernatant was removed from the cells, and the MTT reagent/culture medium mixer (1: 10) was incubated with cells for 3 h. Purple formazan was dissolved in DMSO and then measured using a microplate reader (BioTek; Winooski, VT, USA) at an absorbance of 570 nm.

### 2.3 Wound healing assay

The wound‐healing assay is a method that measures directional cell migration in vitro.^14^ The two wells culture insert (ibidi GmbH; Gräfelfing, Germany) was performed to provide the constant width of the gaps in the cell monolayer. First, place the inserts on the culture surface and seed 3.5 × 10^3^ cells in the reservoirs. After attaching the cell, the inserts were removed, and then the cells were treated with different doses of pyrocurzerenone (0, 25, 50 and 100 μM) for 24 h. The cell migration ability of each group was recorded by micrography. According to the rate of migratory cells, the SCC‐9 cell groups were recorded for 0, 3, 6 and 24 h, while the SAS and SCC‐1 cell groups were recorded for 0, 3 and 6 h.

### 2.4 Transwell migration and invasion assay

The transwell migration and invasion assay is used to measure the capacity of cell motility and invasiveness in vitro.^15^ Briefly, oral cancer cells were treated with different doses of pyrocurzerenone (0, 25, 50 and 100 μM) for 24 h. After placing the transwells (Greiner Bio‐One; Frickenhausen, Germany) in a 24‐well plate with fresh culture medium, cells were counted for 5 × 10^5^ cells/mL and seeded in the upper chamber of the transwells. For the transwell invasion assay, matrigel (BD Biosciences; Billerica, MA, USA) was added to the upper chamber of the transwells before cell seeding. The migration and invasion assay transwells were collected at 24 h, then fixed and stained with 10% Giemsa stain solution (Sigma‐Aldrich; St. Louis, MO, USA). The cell migration and invasion of each group were recorded using micrography.

### 2.5 Western blot assay

A western blot assay was performed to determine protein expression in pyrocurzerenone‐treated oral cancer cells. Cells treated with different doses of pyrocurzerenone (0, 25, 50 and 100 μM) for 24 h were collected and lysed with RIPA buffer (MilliporeSigma) containing protease and phosphatase inhibitors. An equal amount of samples were separated by 10% or 12.5% polyacrylamide gel and then transferred to polyvinylidene fluoride (PVDF) membranes (MilliporeSigma). After blocking with 5% skim milk, membranes were incubated with the indicated primary antibody at 4°C overnight. Primary antibodies were purchased from Cell Signalling Technology (Danvers, MA, USA); primary antibodies against β‐actin were purchased from Novus Biologicals (Centennial, CO, USA). Subsequently, secondary antibodies against rabbit or mouse with peroxidase were incubated with a membrane for 1 h at room temperature. The membranes were measured using a chemiluminescence photometer (ImageQuant LAS 4000 Mini, GE Healthcare Life Sciences; Boston, MA, USA).

### 2.6 Proteome Profiler antibody array

The human protease array kit (R&D Systems Inc., cat. ARY021B; Minneapolis, MN, USA) was conducted to analyse the relative level of selected proteases.^16^ According to the manual, cell lysate were collected and incubated with membranes from the human protease array kit at 4°C overnight. After washing with wash buffer and incubating with streptavidin–HRP solution, membranes were measured using a chemiluminescence photometer (GE Healthcare Life Sciences).

### 2.7 Vector construction and transfection

The pEGFP vector was used to generate pEGFP‐CTSS.^9^ Cells were seeded in a 6‐cm dish and transfected with plasmids using TurboFect Transfection Reagent (Thermo Fisher Scientific; Waltham, MA, USA). The empty vector pEGFP was used as the control group compared to the CTSS over‐expression group. The effects of CTSS overexpression were further detected by western blot assay.

### 2.8 Statistical analysis

The statistical analysis in the present experiments was measured using GraphPad Prism V6.0 (GraphPad Software, Inc.). To compare various groups, one‐way ANOVA followed by Tukey's multiple comparison test is used. The student's *t*‐test is used to compare the two groups. A *p*‐value less than 0.05 is considered significant.

## 3 RESULTS

### 3.1 Effect of pyrocurzerenone on cell viability of oral cancer cells

Oral cancer cells were treated with different concentrations of pyrocurzerenone (0, 25, 50 and 100 μM) for 24, 48 and 72 h and then cell viability using an MTT assay. However, pyrocurzerenone showed no cytotoxic effect in this cell line (Figure 1B–D).

### 3.2 Effect of pyrocurzerenone on oral cancer cell motility

**FIGURE 2:**
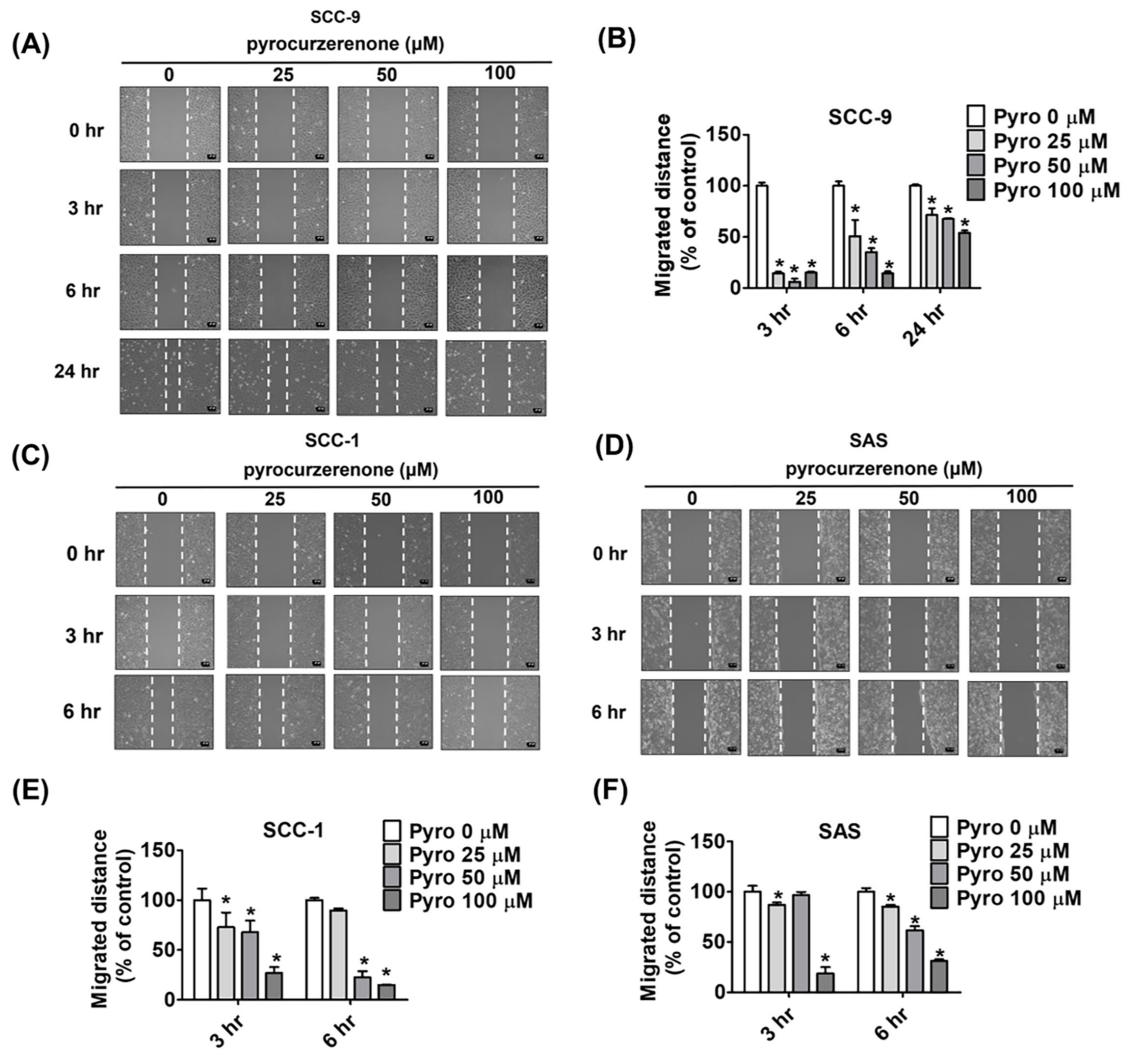
Pyrocurzerenone suppressed cell motility of oral cancer cell lines. (A, C, D) After treatment with different doses of pyrocurzerenone, cell motility was recorded by microphotography at 0, 3, 6 or 24 h. (B, E, F) The number of migrated cells was counted after being treated with pyrocurzerenone for 0 h. Scale bar = 100 μm. * represented *p* < 0.05, compared to the vehicle group. (Pyro, pyrocurzerenone).

**FIGURE 3:**
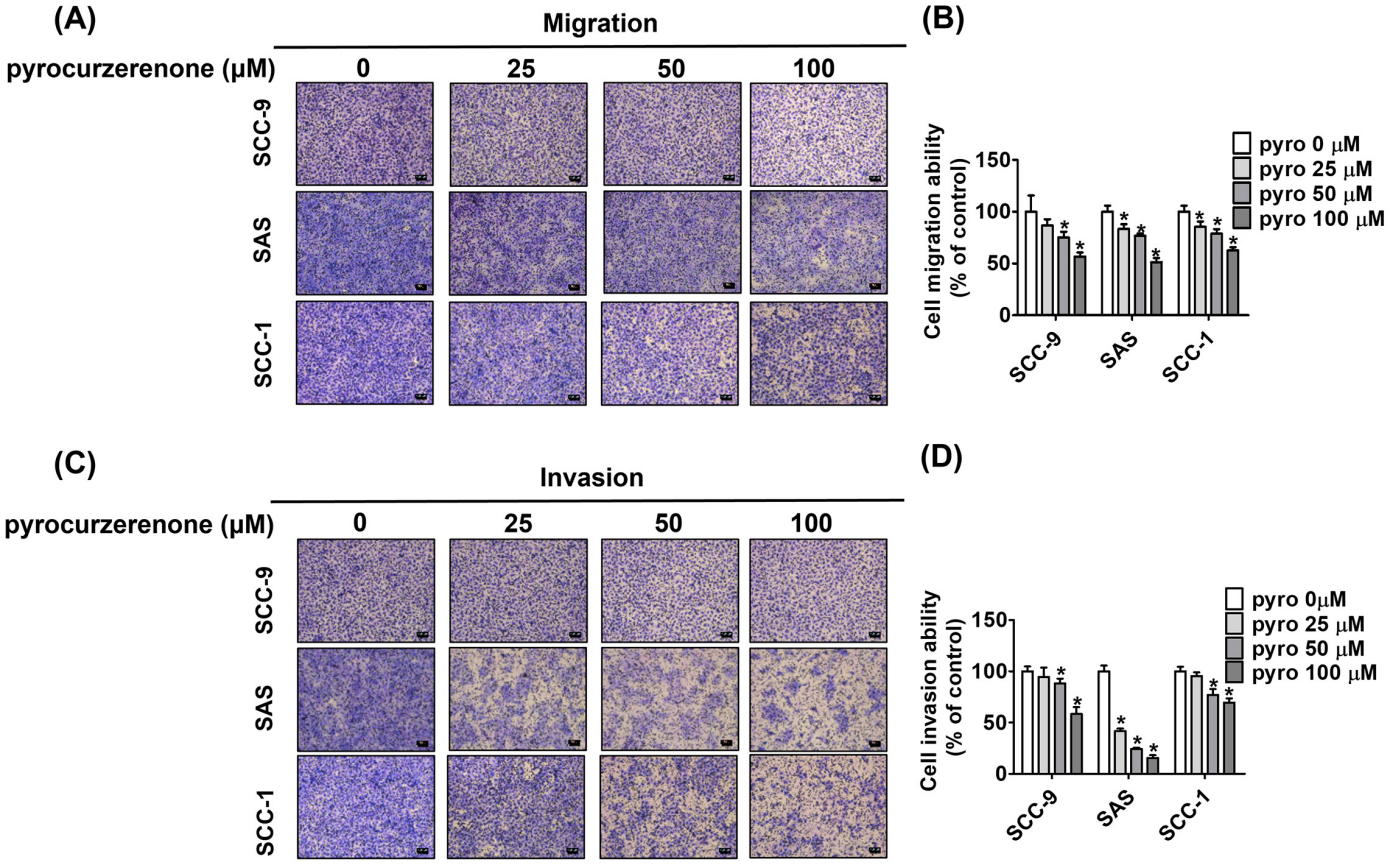
Pyrocurzerenone suppressed migration and invasion of oral cancer cell lines. After treatment with different doses of pyrocurzerenone, cell motility was recorded by microphotography at 24 h. (A, B) The number of migrated cells and (C, D) the number of invaded cells of SCC‐9, SAS, and SCC‐1 cells were counted after being treated with pyrocurzerenone for 24 h. Scale bar = 100 μm. * represented *p* < 0.05, compared to the vehicle group. (Pyro, pyrocurzerenone).

We then performed wound healing and transwell assays to assess cell migration and invasion to determine the capacity of SCC‐9, SCC‐1 and SAS cells to metastasize. By comparing the 24 h results to those of the control group, it was evident that pyrocurzerenone reduced the motility of SCC‐9 cells dose‐dependent (Figure 2A,B). At 6 h, identical results were seen in SAS and SCC‐1 cell lines (Figure 2C,D). In a dose‐dependent way, we could show that pyrocurzerenone greatly limited the capacity of all three cell lines to migrate and invade after 24 h using a transwell test (Figure 3A,B). Collective findings indicate that pyrocurzerenone inhibits cell migration in OSCC cell lines without affecting the cells.

### 3.3 Effect of pyrocurzerenone on the MAPK pathway in oral cancer cell lines

**FIGURE 4:**
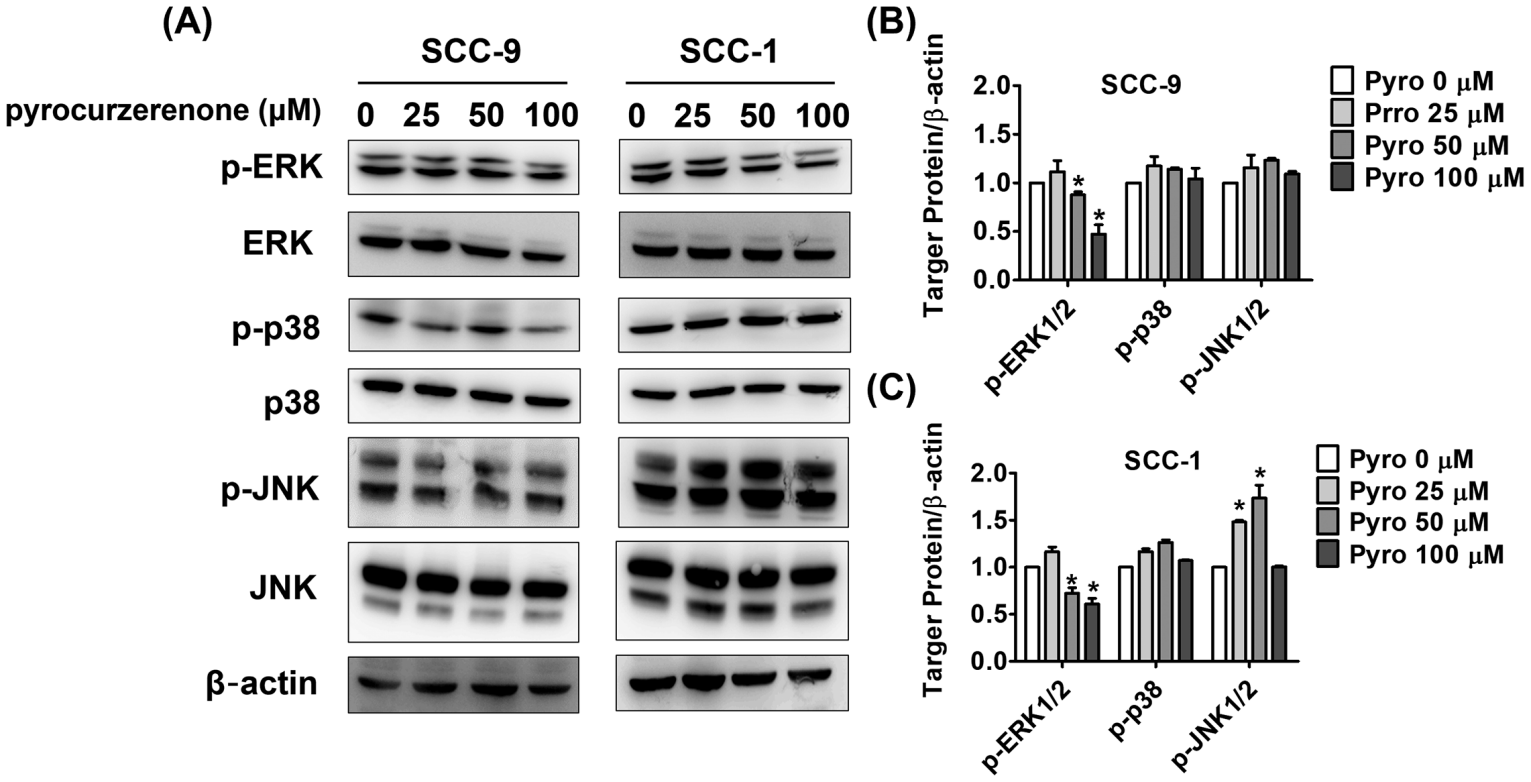
Pyrocurzerenone decreased ERK protein expression in oral cancer cell lines. (A–C) The protein level of ERK, JNK and p38 in SCC‐9 and SCC‐1 cell lines was measured using a Western blot assay, respectively. β‐actin as an internal control was used to adjust protein expression level. * represented *p* < 0.05, compared to the vehicle group.

**FIGURE 5:**
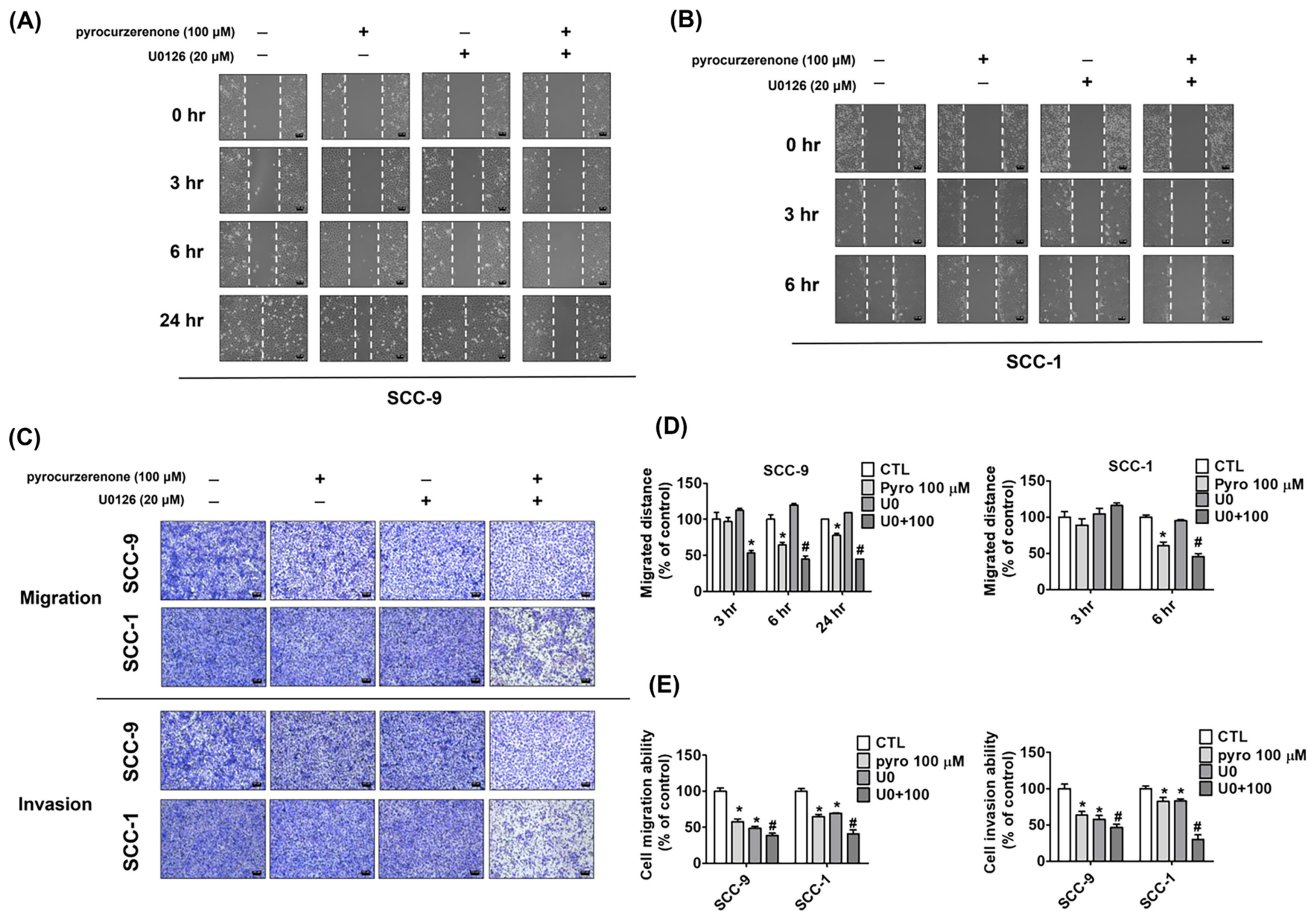
Pyrocurzerenone suppressed cell motility and invasion through the downregulation of ERK in oral cancer cells. (A, B) Cells were pretreated with U0126 for 1 h and followed with or without pyrocurzerenone treatment for 24 h. Subsequently, cell motility was recorded by microphotography at 0, 3, 6 or 24 h. (C–E) The number of migrated and invaded cells was counted after the treatment with U0126, pyrocurzerenone, or both for 24 h. Scale bar = 100 μm. * represented *p* < 0.05, compared to the vehicle group. # represented *p* < 0.05, compared to the pyrocurzerenone (100 μM) treatment group. (CTL, control; Pyro, pyrocurzerenone; U0, U0126).

Using western blot analysis, we further investigated the expression of proteins ERK1/2, JNK1/2 and p38 linked to the MAPK pathway. The findings showed that at 50 and 100 μM, pyrocurzerenone dramatically reduced the amount of ERK phosphorylation in SCC‐9 and SCC‐1 cells (Figure 4A–C). Next, we used pyrocurzerenone treatments (100 μM) in conjunction with the ERK inhibitor U0126 (20 μM) to validate the impact of particular inhibitors on the ERK l/2 pathway in SCC‐9 and SCC‐1 cells. For additional validation, transwell migration and wound‐healing experiments were performed. In SCC‐9 and SCC‐1 cell lines, the ERK inhibitor in combination with pyrocurzerenone treatments (20 μM) considerably increased inhibitory effects on motility (Figure 5A,B), migration and invasion (Figure 5C–E) as compared to the pyrocurzerenone treatment (100 μM) alone.

### 3.4 Pyrocurzerenone downregulated cathepsin S expression

**FIGURE 6:**
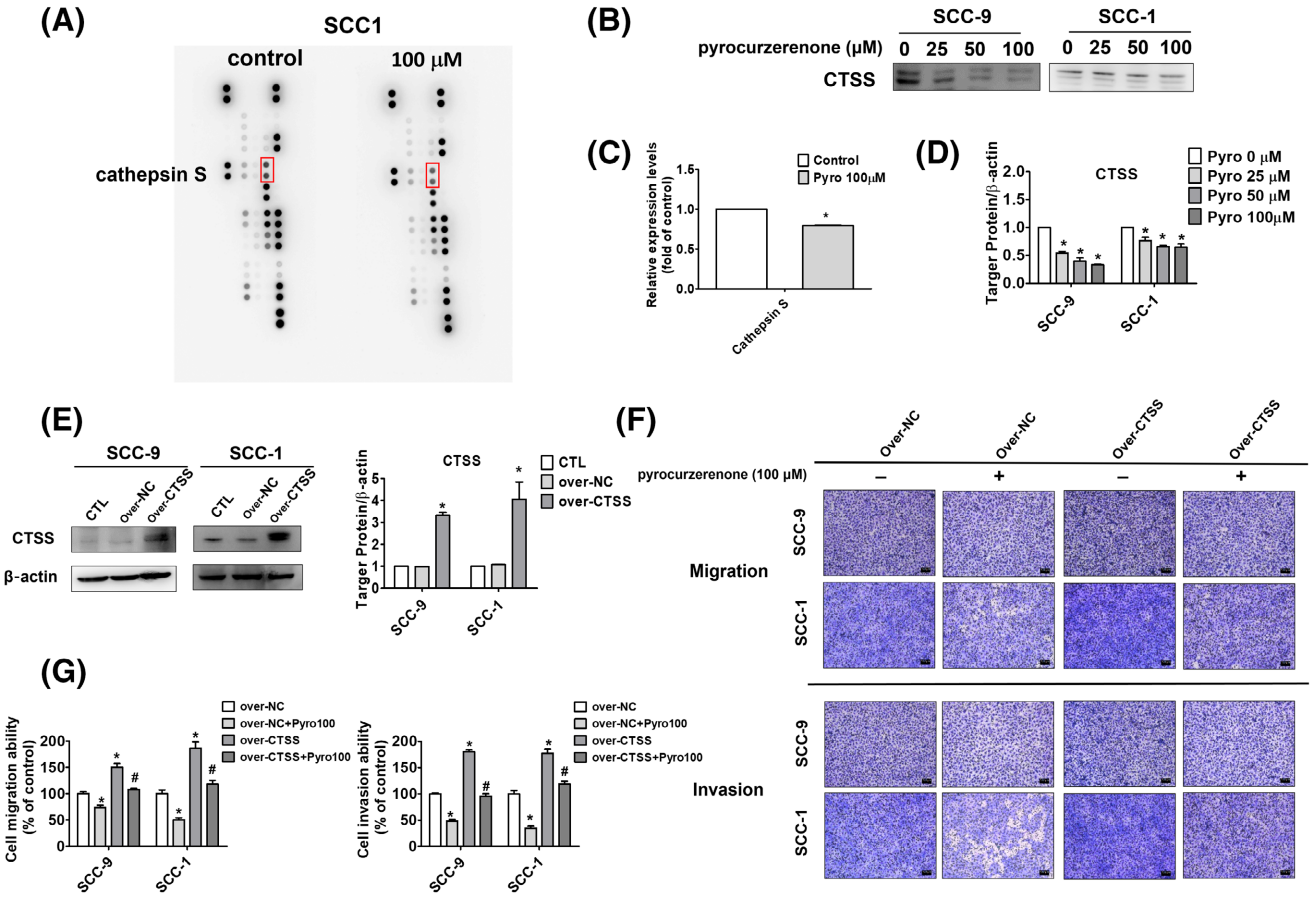
Pyrocurzerenone suppressed cell migration and invasion by decreasing cathepsin S. (A, C) The SCC‐1 cell line was treated with pyrocurzerenone at a dose of 100 μM for 24 h and measured using a proteome profiler antibody array kit. The expression level of cathepsin S was labelled with a grey rectangle. (B, D) The protein expression level of cathepsin S was measured using Western blot after different doses of pyrocurzerenone treatment in SCC‐9 and SCC‐1 cell lines. (E) Cells transfected with overexpression control plasmid or overexpression cathepsin S plasmid were collected to measure the overexpression level of the cathepsin S protein. (F, G) Migration and invasion assays were measured in SCC‐9 and SCC‐1 cells after transfected with overexpression control plasmid or overexpression cathepsin S plasmid followed with or without pyrocurzerenone treatment at 100 μM. Scale bar = 100 μm. β‐actin as an internal control was used to adjust protein expression level. * represented *p* < 0.05, compared to the vehicle group. # represented *p* < 0.05, compared to the pyrocurzerenone (100 μM) treatment group. (CTSS, cathepsin S; over‐NC, overexpression control plasmid; over‐CTSS, overexpression cathepsin S plasmid; Pyro, pyrocurzerenone).

After pyrocurzerenone therapy, the expression level dropped, as seen in Figure 6A,C. SCC‐9 and SCC1 cells were cultured in the presence of increasing doses of pyrocurzerenone for 24 h to validate the effect of pyrocurzerenone on the expression of cathepsin S. The findings demonstrated that the expression of cathepsin S was inhibited by pyrocurzerenone in a concentration‐dependent manner (Figure 6B,D). Additionally, we looked at the possibility that cathepsin S overexpression causes oral cancer cells to migrate. The results reveal that cathepsin S induced migration and invasion in oral cancer cells, while pyrocurzerenone treatment reversed this effect (Figure 6E–G). These findings suggest that pyrocurzerenone inhibited cathepsin S‐induced cell migration and invasion in oral cancer cells.

## 4 DISCUSSION

The current study sought to evaluate the antimetastatic capacity of pyrocurzerenone against three different subtypes of oral squamous cell carcinoma cells. This is the first study demonstrating that pyrocurzerenone has anti‐invasive and anti‐migratory effects in human oral squamous cell carcinoma. Our finding suggests that pyrocurzerenon inhibits metastasis by reducing the activity of ERK and cathepsin S.

To carry out the experiments, we treated SCC‐9, SCC‐1 and SAS cells with different concentrations of pyrocurzerenone (25, 50 and 100 μM) for the indicated time points. According to our data from the MTT experiment used to investigate cytotoxic effects, pyrocurzerenone did not alter the viability of human oral cancer cells for 72 h. Transwell and wound closure assays show that pyrocurzerenone significantly reduces human oral cancer cells' motility, migration and invasion. This leads to decreased cellular ability to migrate and invade.

To explore the molecular mechanism behind the antimetastatic actions of pyrocurzerenone, we considered measuring the activity and quantity of common MAPKs, such as p38, JNK1/2 and ERK1/2. Many studies have demonstrated that activation of MAPK pathways regulates various biological processes, including cell growth, survival, migration, differentiation and apoptosis.^17^, ^18^, ^19^, ^20^ The present study determined that pyrocurzerenone suppresses ERK1/2 in both SCC‐9 and SCC‐1 cells; however, the phosphorylation of the p38 and JNK1/2 proteins increased. The ERK inhibitor U0126 was also used to show that pyrocurzerenone inhibits cell migration by activating ERK. Our findings revealed that U0126 and pyrocurzerenone significantly inhibited cell motility and migration in both the SCC‐9 and SCC‐1 cell lines.

Cathepsin S, a lysosomal cysteine protease, is involved in the inducement of inflammation,^21^, ^22^ migration and invasion.^10^, ^23^, ^24^, ^25^ To identify the protease involved in pyrocurzerenone‐mediated inhibition of metastasis mediated by pyrocurzerenone, a human protease array was performed in SCC‐1 cells. The data showed that pyrocurzerenone markedly reduced the expression of cathepsin S. The western blot assay showed that pyrocurzerenone (0, 25, 50, 100 μM) reduced the expression of cathepsin S dose‐dependent. Cathepsin S was overexpressed in the SCC‐9 and SCC‐1 cell lines and then treated with pyrocurzerenone. Overexpression of cathepsin S increased cell migration and invasion, which was reversed in pyrocurzerenone‐treated oral cancer cells. Our findings demonstrate the crucial role that cathepsin S plays in the impact of pyrocurzerenone on oral cancer. Previous research by Li et al. and Joshi et al. revealed that another non‐canonical mechanism to control metastasis is the ERK pathway.^26^, ^27^, ^28^ In summary, this is the first study to show that pyrocurzerenone reduces the invasiveness of oral cancer by decreasing the production of proteins linked to tumour spread through metastasis.

## AUTHOR CONTRIBUTIONS

**Ming‐Ju Hsieh:** Conceptualization (equal); writing – original draft (lead); writing – review and editing (equal). **Chia‐Chieh Lin:** Methodology (equal); software (equal). **Hsin‐Yu Ho:** Conceptualization (equal); writing – review and editing (equal). **Yi‐Ching Chuang:** Methodology (equal); software (equal). **Yu‐Sheng Lo:** Methodology (equal); software (equal). **Mu‐Kuan Chen:** Conceptualization (equal); writing – review and editing (equal).

## FUNDING INFORMATION

This research did not receive external funding.

## CONFLICT OF INTEREST STATEMENT

The authors confirm that there are no conflicts of interest.

## Data Availability

The data used to support this study's findings are available from the corresponding author upon request.
